# Oxidative stress-induced JNK/AP-1 signaling is a major pathway involved in selective apoptosis of myelodysplastic syndrome cells by Withaferin-A

**DOI:** 10.18632/oncotarget.20497

**Published:** 2017-08-24

**Authors:** Karine Z. Oben, Sara S. Alhakeem, Mary K. McKenna, Jason A. Brandon, Rajeswaran Mani, Sunil K. Noothi, Liu Jinpeng, Shailaja Akunuru, Sanjit K. Dhar, Inder P. Singh, Ying Liang, Chi Wang, Ahmed Abdel-Latif, Harold F. Stills Jr, Daret K. St. Clair, Hartmut Geiger, Natarajan Muthusamy, Kaoru Tohyama, Ramesh C. Gupta, Subbarao Bondada

**Affiliations:** ^1^ Markey Cancer Center and Department of Microbiology, Immunology and Molecular Genetics, University of Kentucky, Lexington, KY 40536, USA; ^2^ Department of Internal Medicine, University of Kentucky, Lexington, KY 40536, USA; ^3^ Comprehensive Cancer Center and Department of Internal Medicine, Ohio State University, Columbus, OH 43210, USA; ^4^ Biostatistics Core, Markey Cancer Center, University of Kentucky, Lexington, KY 40536, USA; ^5^ Division of Experimental Hematology and Cancer Biology, Cincinnati Children's Hospital Medical Center and University of Cincinnati, Cincinnati, OH 45229, USA; ^6^ Department of Toxicology and Cancer Biology, University of Kentucky, Lexington, KY 40536, USA; ^7^ Department of Natural Products, National Institute of Pharmaceutical Research, S.A.S Nagar, Punjab 160062, India; ^8^ Department of Microbiology, Immunology and Molecular Genetics, University of Kentucky, Lexington, KY 40536, USA; ^9^ Department of Laboratory Medicine, Kawasaki Medical School, Kurashiki, Okayama 701-0192, Japan; ^10^ Department of Pharmacology and Toxicology, and James Graham Brown Cancer Center, University of Louisville, Louisville, KY 40202, USA

**Keywords:** myelodysplastic syndrome (MDS), Withaferin A (WFA), apoptosis, JNK/AP-1 signaling, reactive oxygen species (ROS)

## Abstract

Myelodysplastic syndromes (MDS) are a diverse group of malignant clonal hematopoietic stem cell disorders characterized by ineffective hematopoiesis, dysplastic cell morphology in one or more hematopoietic lineages, and a risk of progression to acute myeloid leukemia (AML). Approximately 50% of MDS patients respond to current FDA-approved drug therapies but a majority of responders relapse within 2-3 years. There is therefore a compelling need to identify potential new therapies for MDS treatment. We utilized the MDS-L cell line to investigate the anticancer potential and mechanisms of action of a plant-derived compound, Withaferin A (WFA), in MDS. WFA was potently cytotoxic to MDS-L cells but had no significant effect on the viability of normal human primary bone marrow cells. WFA also significantly reduced engraftment of MDS-L cells in a xenotransplantation model. Through transcriptome analysis, we identified reactive oxygen species (ROS)-activated JNK/AP-1 signaling as a major pathway mediating apoptosis of MDS-L cells by WFA. We conclude that the molecular mechanism mediating selective cytotoxicity of WFA on MDS-L cells is strongly associated with induction of ROS. Therefore, pharmacologic manipulation of redox biology could be exploited as a selective therapeutic target in MDS.

## INTRODUCTION

Myelodysplastic syndromes (MDS) are a diverse group of malignant clonal hematopoietic stem cell disorders characterized by ineffective hematopoiesis, dysplastic cell morphology in one or more hematopoietic lineages, and a risk of progression to acute myeloid leukemia (AML) [1, 2]. Ineffective hematopoiesis manifests clinically as cytopenias – anemia being the most common, bleeding and recurrent infections [1, 3]. Approximately 30% of MDS patients progress to AML while progressive disease in the other 70% culminates in complete bone marrow failure [4]. About 15% of MDS cases occur as a late complication of exposure to cytotoxic therapy and are classified as therapy-related MDS (t-MDS) [1]. MDS are generally thought of as diseases of the elderly, with a median age at diagnosis of 65-70 years [1]. However, the age of diagnosis for t-MDS correlates with the age of cytotoxic therapy treatment [5].

MDS is incurable with current FDA-approved drug therapies (azacitidine, decitabine and lenalidomide). Approximately 50% of MDS patients respond to these therapies and a majority of responders relapse within 2-3 years [6–8]. Patient outcomes after drug therapy failure are very poor with median overall survival and 2-year survival probability of 5.6 months and 15%, respectively [8–10]. Hematopoietic stem cell transplantation (HSCT) is potentially curative but comorbidities and treatment-related morbidity and mortality in older patients limits its use; less than 10% of MDS patients are referred to HSCT [11]. Given the current status of MDS treatment, there is a compelling need to investigate potential new therapies for MDS treatment.

Scientific exploration of plant-derived compounds for cancer treatment is increasing because they are thought to be less toxic than current chemotherapy and radiotherapy regimens [12]. Withaferin A (WFA) is a plant-derived steroidal lactone isolated from *Withania somnifera* (also known as Indian Ginseng, Indian Winter cherry or Ashwagandha) with demonstrated anticancer activities in several cancer models including prostate, breast, cervical and pancreatic cancers, as well as melanoma and lymphoma [13–15].

The heterogeneity of MDS has made it difficult to generate a mouse that models complete disease phenotype, and xenotransplantation of patient bone marrow cells into immunocompromised mice is poor and highly inefficient [16, 17]. We utilized the validated human MDS-L cell line, which has been used to successfully establish a MDS xenograft model [18–20], to determine if the anticancer effects of WFA extend to MDS. Our data demonstrate that WFA induces selective cytotoxicity of MDS-L cells while sparing normal bone marrow cells both *in vitro* and *in vivo*. Biochemical studies identified reactive oxygen species (ROS) induced JNK/AP-1 apoptotic cell death as a major pathway by which WFA causes cytotoxicity in MDS-L cells.

## RESULTS

### WFA has a potent but selective anti-proliferative effect on MDS-L cells *in vitro* and *in vivo*

**Figure 1:**
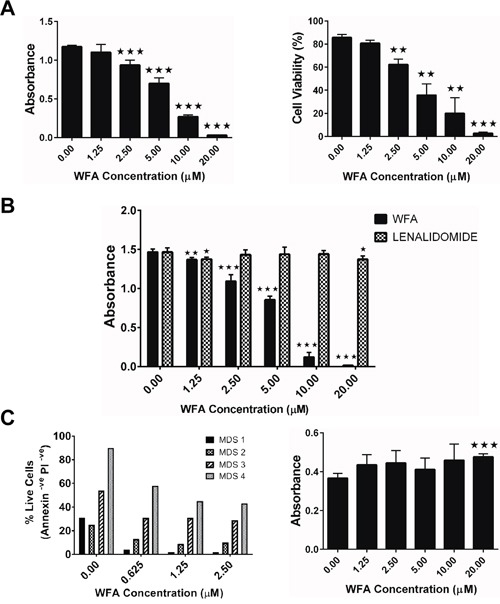
WFA selectively suppresses survival of MDS-L and human primary MDS patient bone marrow cells *in vitro* **(A)** MDS-L cells were treated with increasing concentrations of WFA for 48 h and cell viability was measured by MTT assay (*left*) or trypan blue exclusion (*right*). Data are presented as mean ± SD of triplicate cultures. Results from one (n>3) of similar experiments are shown. **(B)** MDS-L cells were treated with increasing concentrations of WFA or LENA for 48 h and cell viability was determined by MTT assay. Presented data are mean ± SD of triplicate cultures and are representative of three independent experiments. **(C)** Human primary MDS patient bone marrow samples were treated with varying concentrations of WFA for 24 h and cell viability was assessed by annexin-V/PI assay (*left*). Cell viability of normal human primary bone marrow cells treated with WFA for 48 h was measured by MTT assay (*right*). Presented are mean ± SD of triplicate cultures and are representative of two independent experiments. ** = p<0.005, *** = p<0.0005 indicates statistically significant differences between treated and control values.

The MDS-L cell line is not only a validated *in vitro* model for MDS but is also representative of highly aggressive disease, displaying deletions in chromosomes 5 and 7 [18, 19]. These deletions are the most common cytogenetic abnormalities observed in MDS and are associated with significantly worse prognosis [21–23]. Initial studies showed that WFA inhibited *in vitro* proliferation of MDS-L cells in a dose dependent manner (Figure 1A), with an IC_50_ in the 6-9μM range. The decrease in MDS-L cell proliferation by WFA was accompanied by a decrease in cell viability (Figure 1A), which was also dose-dependent. Lenalidomide (LENA) is the FDA-approved treatment for MDS subjects harboring a deletion in chromosome 5q (del (5q)) [24]. Since MDS-L cells have a deletion in chromosome 5 [19], we assessed the relative efficacy of WFA in comparison to LENA. Notably, WFA was substantially more effective than LENA in inhibiting MDS-L cell proliferation *in vitro* (Figure 1B). The modest cytotoxicity of LENA on MDS-L cells we observed (Figure 1B) was in contrast to reported studies [19]. Therefore, we replicated the reported cytotoxic effects of LENA on MDS-L cells [19] by showing that LENA treatment every 24 h inhibited MDS-L proliferation (Supplementary Figure 1A). Although LENA caused some cell death over time (cell viability dropped from 90% to 50% by day 9) (Supplementary Figure 1B), the number of cells recovered at each time point was the same or slightly higher than the number seeded. These observations suggested that LENA had more of a cytostatic effect on MDS-L cells compared to WFA, which was more cytotoxic.

The clinical relevance of the MDS-L data was validated by showing that WFA induced apoptotic cell death of human primary bone marrow cells from MDS patients (Figure 1C). Control experiments with normal human primary bone marrow cells showed no appreciable toxicity at all the doses tested (Figure 1C), demonstrating that the cytotoxic effects of WFA are selective to malignant MDS cells.

**Figure 2:**
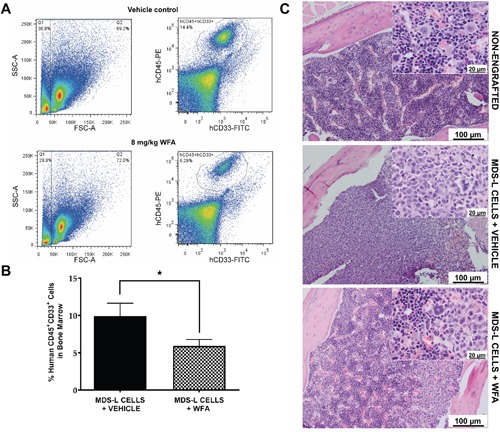
WFA significantly reduces engraftment of MDS-L cells in the bone marrow of NSGS mice **(A)** Representative flow cytometry profiles of vehicle or WFA treated mice using the gating scheme illustrated in Supplementary Figure 2B. **(B)** Average MDS-L bone marrow engraftment of 20 mice in the vehicle control group and 27 mice in the WFA group ± SD. * = p<0.05. **(C)** Representative hematoxylin and eosin staining of paraffin-embedded bones from non-engrafted mice or engrafted mice treated with vehicle or 8 mg/kg WFA.

We utilized the MDS-L NSG-hSCF/hGM-CSF/hIL3 (NSGS) xenograft model [20] to investigate the anti-MDS effect of WFA *in vivo* by comparing MDS-L bone marrow engraftment in vehicle versus WFA-treated mice (Supplementary Figure 2A, 2B). WFA (8 mg/kg) significantly reduced bone marrow engraftment of MDS-L cells in NSGS mice compared to the vehicle treatment (Figure 2A, 2B). Immunohistochemical examination of sample bone marrow tissues confirmed a more prominent uniform infiltrate of cells with displacement of the normal hematopoietic cell population in vehicle-treated engrafted mice, but WFA treatment restored the marrow of engrafted mice to a more normal appearance, with all hematopoietic components in varying stages of maturation (Figure 2C). Remarkably, WFA treatment did not cause any apparent bone marrow suppression of endogenous mouse stem cells (Supplementary Figure 2C). This is of particular importance because chemotherapeutic drugs generally cause bone marrow suppression, which leads to treatment delays and significant dose reductions [25]. These experiments indicate that WFA has an anti-proliferative effect on MDS-L cells both *in vitro* and *in vivo* without exerting non-specific toxicity to normal cells.

### WFA induced apoptosis of MDS-L cells

NF-κB has been implicated in hematologic malignancies and is a suggested potential therapeutic target in MDS [26]. Despite reported ability of WFA to target NF-kB in lymphoma models [14], microscopy analyses revealed WFA treatment did not alter subcellular distribution of NF-κB in MDS-L cells (Supplementary Figure 3A, 3B). Western blot analyses confirmed that NF-κB nuclear/cytoplasmic distribution is unaltered by WFA treatment in MDS-L cells (Supplementary Figure 3C), indicating that WFA inhibits proliferation of MDS-L cells by NF-κB independent mechanism(s).

**Figure 3:**
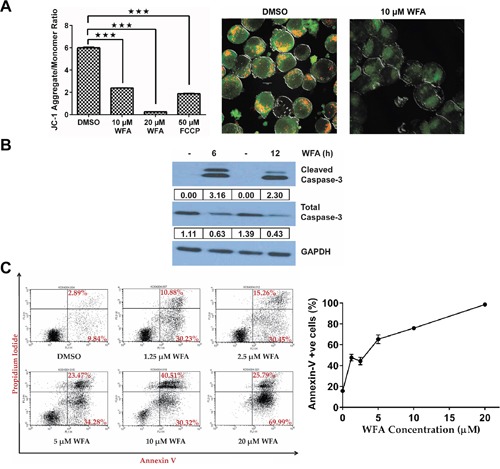
Effect of WFA on MMP, CASPASE-3 activation and annexin-V/PI staining in MDS-L cells **(A)** The effect of WFA treatment (10 μM or 20 μM for 8 h) or FCCP (50 μM for 2 h) on MMP in MDS-L cells was determined by JC-1 assay. The distribution of JC-1 aggregates (red) and monomeric JC-1 (green) was analyzed by flow cytometry. Mean ratios ± SD of JC-1 aggregates/monomers as a function of treatment (n = 3), where *** = p<0.0005 (*left*). Representative data from one of multiple experiments (n>3) with similar results are shown. Microscopy images showing changes in mitochondrial JC-1 accumulation of MDS-L cells after 8 h of 10 μM WFA treatment (*right*). **(B)** Immunoblots for total and cleaved CASPASE-3 expression with DMSO or WFA treatment (6 or 12 h) in MDS-L cells. Expression normalized to GAPDH is indicated. Results are representative of three experiments. **(C)** MDS-L cells were treated with increasing concentrations of WFA for 48 h and apoptosis assessed by annexin-V/PI staining. Representative flow cytometry profiles by WFA concentration (*left*) and the percentage of annexin-V positive cells at different doses of WFA are presented as mean ± SD (n = 3) (*right*). Data are representative of three independent experiments.

We utilized gene expression changes induced by WFA treatment to query the pathway(s) regulated by WFA in MDS-L cells, and focused on early time points (6 and 12 h) to identify primary gene alterations. Shown in Supplementary Figure 4, gene set enrichment analysis (GSEA) of differentially expressed genes (fold change > 3 and q-value <0.05) between WFA treatment and DMSO groups revealed a highly significant enrichment in expression of apoptosis-related genes by WFA at both 6 h (false discovery rate (FDR) q-value = 0.0001; family-wise error rate (FWER) p-value = 0.0001) and 12 h (FDR q-value = 0.0001; FWER = 0.008). So we investigated if WFA decreased mitochondrial membrane potential (MMP) in MDS-L cells, as it is a well-known indicator of apoptosis [27]. MMP was assessed by the widely used JC-1 assay, which measures changes in the ratio of JC-1 mitochondrial aggregates to cytoplasmic monomers [28]. Flow cytometric analysis revealed a decrease in JC-1 aggregates with a concomitant increase in JC-1 monomers in WFA-treated MDS-L cells compared to DMSO control (Supplementary Figure 5), indicating a decrease in MMP in MDS-L cells with WFA treatment (Figure 3A). Microscopy analysis confirmed that accumulation of JC-1 aggregates was significantly lower in WFA-treated MDS-L cells relative to DMSO treatment (Figure 3A), consistent with a decrease in MMP.

During apoptosis, a decline in MMP ultimately results in the activation of caspases by proteolytic cleavage, a hallmark of apoptosis [27, 29]. Of the known human caspases, CASPASE-3 is the most frequently activated that commits cells to apoptosis [29]. WFA significantly activated CASPASE-3 in MDS-L cells in as early as 6 h and the activated protease persisted for at least 6 more hours (Figure 3B). As expected, we observed a simultaneous decrease in total CASPASE-3 (Figure 3B). In accordance with the MMP and caspase-3 data, WFA caused a dose-dependent increase in annexin-V positive apoptotic cells (Figure 3C).

### Treatment with WFA activates JNK/AP-1 signaling in MDS-L cells

We next sought to identify the critical pathway(s) mediating the anti-proliferative effects of WFA on MDS-L cells. The finding that growth suppression of MDS-L cells by WFA involved cell death by apoptosis (Figure 3) narrowed our focus to pro-apoptotic signaling pathways that involve major WFA-regulated genes in MDS-L cells. Shown in Supplementary Figure 6 are the top 10 elevated and repressed genes identified by microarray analysis, arranged according to average fold change. *JUN* and *FOSB* were among the top 3 up-regulated genes at 6 h (47 and 36 fold respectively) and the top 2 up-regulated genes at 12 h (52 and 60 fold respectively). C-JUN and FOSB heterodimerize to form an AP-1 transcription factor that is activated by phosphorylation of the C-JUN subunit by JNK, a signaling pathway that has been demonstrated to regulate apoptosis [30]. A receptor-mediated event or oxidative stress is usually associated with activation of the JNK/AP-1 signaling pathway [30]. Since ROS production had been implicated in the anti-cancer effects of WFA in other systems, we hypothesized that WFA activates ROS-mediated JNK/AP-1 signaling in MDS-L cells.

**Figure 4:**
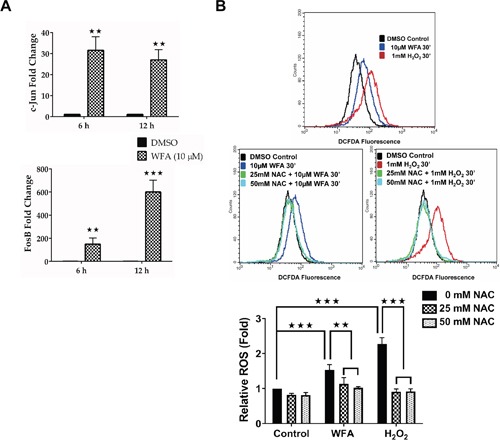
WFA treatment increased C-JUN/FOSB expression and ROS production in MDS-L cells **(A)** qRT-PCR analysis of C-JUN (*top*) and FOSB (*bottom*) expression in cells treated with DMSO or WFA (6 or 12 h) using human specific primers. Gene amplification was normalized to RPII expression and relative amplification was determined by normalizing to DMSO control. ** = p<0.005, *** = p<0.0005. Presented data are representative of two independent experiments. **(B)** Representative flow cytometry profiles of ROS production in MDS-L cells by DCFDA fluorescence showing ROS production in MDS-L cells exposed to 10 μM WFA for 30 min (*top*), in the presence or absence of NAC (middle). Amount of ROS produced normalized to DMSO control samples is presented as mean ± SD (n = 3) (*bottom*). ** = p<0.005, *** = p<0.0005. Data are representative of three experiments.

qRT-PCR confirmed a robust increase in C-JUN and FOSB mRNA in MDS-L cells treated with WFA (Figure 4A). Next, we investigated if WFA treatment caused any change in ROS production in MDS-L cells, since ROS has been implicated in activation of JNK and the downstream AP-1 pathway [31]. WFA increased ROS accumulation in MDS-L cells compared to DMSO as measured by increased fluorescence of the ROS sensitive dye, carboxy-H_2_DCFDA (Figure 4B). Pretreatment with the ROS scavenger, N-acetyl cysteine (NAC), 4 h prior to WFA treatment led to complete inhibition of WFA-induced ROS (Figure 4B).

**Figure 5:**
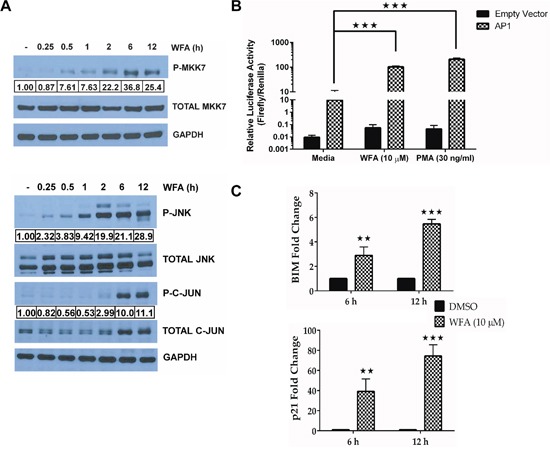
JNK/AP-1 signaling is activated in WFA-treated MDS-L cells **(A)** Phospho and total protein immunoblots for MKK7, JNK and C-JUN in WFA-treated (10 μM) MDS-L cells for the indicated time-points. Activated protein expression levels normalized to the respective total protein are presented. Data are representative of at least two independent experiments. **(B)** MDS-L cells co-transfected with either an AP-1 or empty firefly luciferase expression vector and a renilla luciferase vector under the control of a constitutive promoter (2:1) were treated with WFA (10 μM) or PMA (30 ng/ml) for 12 h and promoter activity was assessed by the dual Glo luciferase assay. Firefly luciferase activity relative to renilla luciferase activity is shown as mean ± SD of triplicate cultures. Presented data are representative of three independent experiments. **(C)** BIM (*top*) and p21 (*bottom*) mRNA expression was evaluated by qRT-PCR in MDS-L cells treated with WFA. Gene amplification was normalized to RPII expression and relative amplification was determined by normalizing to DMSO control. ** = p<0.005, *** = p<0.0005. Presented data are representative of two experiments.

The ROS sensitive MAP3K, apoptosis signal-regulating kinase 1 (ASK1), mediates JNK signaling by phosphorylating and activating MKK7 [31, 32]. Therefore, we sought to determine the level of MKK7 phosphorylation in MDS-L cells after WFA treatment. WFA increased phosphorylation of MKK7 (Figure 5A). MKK7 is a MAP2K known to specifically activate JNK [33], which in turn phosphorylates nuclear c-Jun [30]. Increased phosphorylation of both JNK and C-JUN was observed in WFA-treated MDS-L cells (Figure 5A). The fact that C-JUN phosphorylation was only detected at 6 h compared to JNK phosphorylation, which was detected as early as 30 min post-treatment, suggested a sequential activation of the signaling cascade. Total C-JUN protein levels increased at both 6 and 12 h (Figure 5A) which was in agreement with the qRT-PCR results (Figure 4A). These results suggest that both the increase in JNK activation and C-JUN expression could be contributing to the observed increase in C-JUN phosphorylation.

We next investigated the potential functional relationship between increased C-JUN phosphorylation and the transcriptional activity of AP-1. MDS-L cells transfected with either an AP-1 reporter or the empty vector expressing firefly luciferase were treated with WFA for 12 h, and promoter activity assessed by the dual Glo luciferase assay. The increase in C-JUN phosphorylation led to an increase in AP-1 activity since WFA-induced AP-1 promoter driven luciferase activity in MDS-L cells was comparable to phorbol myrsitate acetate (PMA) (Figure 5B), the known AP-1 activator [34]. Increase in AP-1 transcriptional activity was further demonstrated by WFA-induced increase in BIM (BCL2L12) and p21 mRNA expression (Figure 5C), both of which are bona fide AP-1 targets [35, 36].

### JNK/AP-1 signaling is a significant mediator in apoptosis of MDS-L cells by WFA

**Figure 6:**
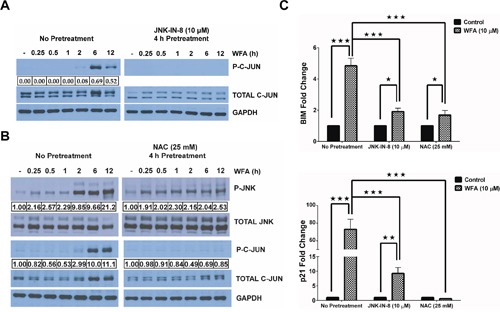
WFA-induced ROS mediates JNK/AP-1 signaling **(A)** Western blot analysis of phospho and total C-JUN protein levels in MDS-L cells treated with JNK-IN-8. MDS-L were cells treated with WFA for the indicated time-points either with or without pretreatment with JNK-IN-8 (10 μM) for 4 h. Phospho-C-JUN expression relative to total C-JUN is presented. **(B)** Immunoblots of phospho- and total protein levels for JNK and C-JUN showing the effect of ROS blockade by a 4 h pretreatment with NAC (25 mM) on WFA-induced JNK activation in MDS-L cells at the indicated time points. Phospho-JNK or phospho-C-JUN protein expression is shown normalized to the respective total protein. **(C)** BIM (top) and p21 (bottom) mRNA expression by qRT-PCR in WFA-treated MDS-L cells with or without prior JNK inhibition (by 4 h pretreatment with 10 μM JNK-IN-8 or 25 mM NAC). Gene amplification was normalized to RPII expression and relative amplification was determined by normalizing to DMSO control. * = p<0.05, ** = p<0.005, *** = p<0.0005.

**Figure 7:**
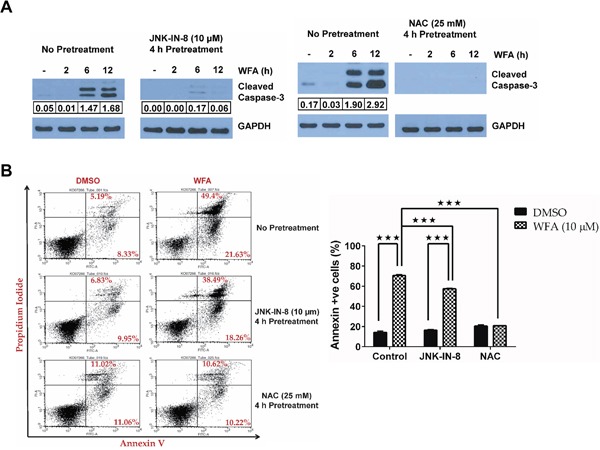
JNK/AP-1 signaling mediates apoptosis in WFA-treated MDS-L cells **(A)** Western blot analysis of active CASPASE-3 (cleaved CASPASE-3) and GAPDH levels in MDS-L cells treated with WFA for 2, 6 or 12 h, with or without JNK blockade by 10 μM JNK-IN-8 (left) or 25 mM NAC (right) pretreatment for 4 h. Cleaved CASPASE-3 expression normalized to GAPDH is shown. **(B)** MDS-L cells with or without inhibitor pretreatment (JNK-IN-8 or NAC for 4 h) were treated with WFA (10 μM) for an additional 24 h and stained with annexin-V and PI. Representative flow cytometry profiles of annexin-V/PI staining (*left*). Frequency of annexin-V positive cells are presented as mean ± SD (n = 3) (*right*). *** = p<0.0005. Data from one of two similar experiments are shown.

To address the importance of JNK signaling in WFA-induced apoptosis of MDS-L cells, we employed a well-characterized selective covalent JNK inhibitor [37], JNK-IN-8, and the well-known antioxidant NAC.JNK-IN-8 pretreatment inhibited WFA-induced C-JUN phosphorylation (Figure 6A). Induction of ROS by WFA is upstream of JNK activation because NAC pretreatment significantly inhibited WFA-induced JNK activation (Figure 6B). Moreover, phosphorylation of C-JUN, JNK's downstream target, was also substantially inhibited by pretreatment with NAC (Figure 6B). Consequently, there was a decrease in AP-1 transcription in MDS-L cells pretreated with either JNK-IN-8 or NAC, compared to WFA-only treated cells as demonstrated by the significant downregulation in transcription of AP-1 targets, BIM and p21 (Figure 6C). JNK/AP-1 signaling is important for apoptosis of WFA-treated MDS-L cells because we observed a substantial decrease in WFA-induced CASPASE-3 activation upon pretreatment with JNK-IN-8 or NAC (Figure 7A).

Off-target effects are always a concern with the use of small-molecule inhibitors in biological systems. To address this issue, we used another widely used reversible ATP-competitive JNK inhibitor, SP600125 [38]. Similar results with a second inhibitor would argue against the possibility that the phenotype observed with JNK-IN-8 was due to off-target effects, since SP600125 is likely to have a different spectrum of off-targets. As was observed with JNK-IN-8, SP600125 also decreased WFA-induced JNK activation (Supplementary Figure 7A) and caspase-3 activation (Supplementary Figure 7B).

### Selective killing of MDS-L cells by WFA is mediated by ROS

**Figure 8:**
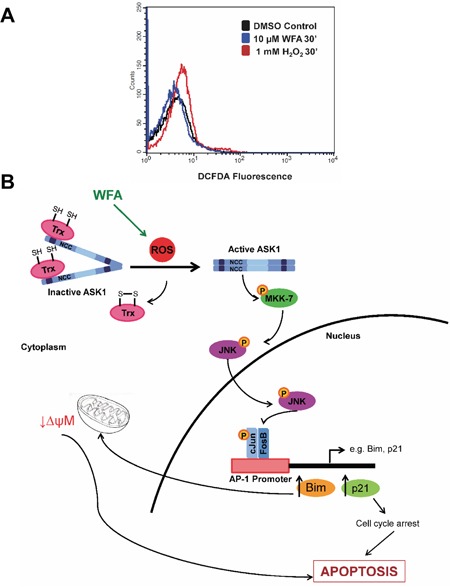
The role of ROS induction in WFA-mediated apoptosis signaling in MDS **(A)** WFA-induced (10 μM, 30 min) ROS production in normal human primary bone marrow cells by flow cytometry analysis of DCFDA fluorescence. **(B)** Model depicting the role of ROS in WFA-mediated cytotoxicity to MDS cells via JNK/AP-1 activation. As illustrated, WFA treatment increases ROS which activates JNK/AP-1 signaling. Increase in expression of AP-1 target genes leads to direct and indirect activation of apoptosis.

The significant contribution of JNK/AP-1 signaling in apoptotic cell death of MDS-L cells was corroborated by a reduction in annexin-V/PI positive cells upon JNK inhibition with JNK-IN-8 in WFA-treated cells (Figure 7B). Interestingly, NAC pretreatment completely protected MDS-L cells from apoptosis triggered by WFA treatment (Figure 7B). These results suggest that not only is ROS upstream of JNK/AP-1 signaling activation (Figure 6), it is the predominant mediator by which WFA induced cytotoxicity in MDS-L cells. This suggestion was substantiated by the observation that WFA failed to increase ROS in normal human primary bone marrow cells (Figure 8A) which were resistant to WFA-induced cell death (Figure 1C). Induction of oxidative stress therefore, likely mediates the selective cytotoxicity of WFA to MDS-L cells while sparing normal bone marrow cells *in vitro* and *in vivo*.

## DISCUSSION

The responses to current drug treatments for MDS is poor [6–8]. Hence, there is a dire need to investigate new therapeutic options. In this study, we demonstrated that the plant-derived steroidal lactone isolated from *Withania somnifera*, WFA, is selectively cytotoxic to MDS-L cells both *in vitro* and *in vivo*. Using gene expression changes induced by WFA and systematic biochemical analysis, we identified ROS-activated JNK/AP-1 signaling as a major pathway through which WFA induces MDS-L apoptosis. The efficiency with which NAC prevented WFA-induced apoptosis of MDS-L cells and the failure of WFA to induce oxidative stress in normal human primary bone marrow cells suggest that the molecular mechanism mediating selective cytotoxicity of WFA on MDS-L cells is associated with induction of ROS. This was supported by the observation that WFA does not induce cell death in normal human primary bone marrow cells but induces apoptosis of MDS patient-derived primary bone marrow cells.

For the first time, our studies established a link between WFA-induced ROS, JNK/AP-1 signaling and apoptosis in MDS-L cells. WFA-induced AP-1 activation was demonstrated by an increase in the mRNA expression of bona fide AP-1 targets [35, 36], BIM and p21. BIM is a crucial apoptosis regulator [39, 40] which induces BAX and BAD activation by inhibiting anti-apoptotic proteins such as BCL-2 and MCL-1; these activities result in increased mitochondrial permeability and apoptosis [41]. WFA caused a significant decrease in MMP in MDS-L cells. Cell cycle inhibitor, p21, could also contribute to WFA-induced apoptosis of MDS-L cells since prolonged cell cycle arrest triggers apoptosis [36]. Cell cycle analysis by flow cytometry showed WFA did induce MDS-L cell arrest at both S and G2/M phases (Supplementary Figure 8A). Consistent with these cell cycle analysis data, treatment with WFA significantly decreased mRNA and protein levels of the cyclins and CDKs required to complete both S (cyclin A2, CDK2) and G2/M (cyclin B1, CDK1) phases of the cell cycle (Supplementary Figure 8B, 8C). Apoptosis analyses by both CASPASE-3 expression and annexin-V staining revealed JNK/AP-1 signaling to be a major pathway activated by increased ROS which mediates apoptosis in WFA-treated MDS-L cells. The lack of strict correlation between the CASPASE-3 and annexin-V assays could be due to lack of binding of the inhibitor to newly synthesized JNK protein. This is a likely possibility as JNK inhibition by JNK-IN-8 occurs via the formation of an irreversible covalent bond with a conserved cysteine residue [37], although complete gene deletion studies are required for confirmation. Taken together, we propose a model by which WFA increases ROS in MDS-L cells to induce cell death primarily by apoptosis via the JNK/AP-1 pathway (Figure 8B).

Redox homeostasis is an essential albeit delicate balance as excess ROS can result in apoptotic cell death [31]. Most cancer cells are at a higher oxidative state than their normal counterparts [42–44] thus, an increase in ROS would therefore push cancer cells beyond the toxic threshold [44, 45]. It is known that cancer cells adapt to this increased oxidative state by upregulating their antioxidant capacity, which decreases their ability to regulate further changes in oxidative stress compared to normal cells [44, 46]. Upregulation of antioxidants as an adaptation to intrinsic oxidative stress is a phenomenon that has been demonstrated to be true in MDS [47, 48]. The higher oxidative state of cancer cells therefore, provides a target for therapeutic selectivity [44]. Several studies have demonstrated the potential of exploiting redox biology differences between malignant and normal cells in discriminately eradicating malignant cells with minimum toxicity to normal cells [49]. Although targeting redox biology is promising therapeutic strategy, one of the challenges is to determine the optimal drug dose that pushes malignant but not normal cells beyond the toxic threshold. A recent report suggested that a 1 μM dose of WFA causes limited apoptosis of MDS-L cells, likely due to simultaneous induction of both autophagy and apoptosis [50] but our studies demonstrated that a 10 μM dose induced robust apoptotic cell death in MDS-L cells. The variable outcomes could be due to differences in dose or purity of WFA used. Identifying the optimal dose of prooxidants is therefore, a critical step for the development of such agents as cancer therapeutics.

A major goal in cancer therapy is to develop drugs that selectively target malignant cells with minimum toxicity to normal cells. The implication of oxidative stress in the development and prognosis of MDS [47] suggests ROS-inducing agents could eradicate MDS cells. Our studies indicate that the growth-suppressive effects of WFA on MDS are mediated by prooxidant mechanisms that discriminate malignant cells from normal cells. This is of particular importance because chemotherapeutic drugs generally cause bone marrow suppression which leads to treatment delays and significant dose reductions [25]. Some MDS patients progress to AML and several ROS-inducing agents are cytotoxic to AML cells [4, 49]. Interestingly, we found that WFA was effectively cytotoxic to both KG1 AML cell line and primary human AML cells (Supplementary Figure 9) suggesting it has therapeutic potential across the MDS disease progression spectrum, as well as in AML. Our finding that the selective cytotoxicity of WFA on MDS-L cells is strongly associated with induction of ROS, justify targeting oxidative stress as a selective therapeutic approach in MDS.

## MATERIALS AND METHODS

### Reagents

WFA was isolated from *Withania somnifera* extract (Sabinsa Corp) using a series of solvent extractions and silica gel-based vacuum liquid column chromatography at the University of Louisville and at the laboratory of Dr. I. P. Singh, National Institute of Pharmaceutical Education Research (NIPER), India. The purity was found to be >94% by UPLC. WFA was reconstituted in dimethyl sulfoxide (DMSO). Anti-human CD45-PE (12-9459-42) and CD33-FITC (11-0339-42) were purchased from eBioscience (San Diego, CA). Cremophor (C5135), Carbonyl cyanide 4-(trifluoromethoxy)phenylhydrazone (FCCP) (C2920), hydrogen peroxide (H_2_O_2_) (H1009), Phorbol 12-myristate 13-acetate (PMA) (P1585), N-acetyl-cysteine (NAC) (A8199), RNase A (R6513), SP600125 (S5567), JNK-IN-8 (SML-1246), JC-1 (T4069), HEPES solution (H0887), ethylenediaminetetraacetic acid (EDTA) solution (E7889), dimethyl sulfoxide (D2438) and monoclonal anti-β-actin antibody (A5441) were purchased from MilliporeSigma-Aldrich (St. Louis, MO). Antibodies to P-p38 (9211S), total p38 (9212), cleaved caspase-3 (9661S), total caspase-3 (9665S), P-MKK7 (4171S), P-c-Jun (9261S), total c-Jun (9162), GAPDH (2118S), CDK2 (2546P), cyclin A (4656P), CDK1 (9116) and cyclin B (4135) were obtained from Cell Signaling Technology (Danvers, Massachusetts). Anti-total MKK7 was purchased from Zymed (32-7000). Antibodies to Hdac1 (7872), NF-κB p65 (372) P-JNK (6254) and total JNK (571) and NF-κB p65 (372) were obtained from Santa Cruz Biotechnology (Santa Cruz, CA). Peroxidase coupled goat anti-rabbit (SC-2004) and anti-mouse (SC-2005) Ig secondary antibodies were also acquired from Santa Cruz Biotechnology (Santa Cruz, CA). 1X phosphate buffered saline (16750-078) was obtained from VWR (Radnor, PA). Lenalidomide (NC0600901) was purchased from Thermo Fisher Scientific (Waltham, MA). Biotin conjugated rat anti-CD45R/B220 (553086), anti-CD11b (553309), anti-Gr-1 (553125), anti-CD8α (5532029), anti-Ter-119 (553672), anti-CD5 (553019); streptavidin APC CY7 (554063) and anti c-KIT-APC (553356) were purchased from BD Pharmingen (San Diego, CA). Anti-Sca-1-PB (122520) was purchased from BioLegend (San Diego, CA).

### Cell culture

MDS-L cells were maintained in IMDM/Ham's F-12 (50:50) medium supplemented with 12% fetal bovine serum (Atlanta Biologicals), 5 μg/ml apotransferrin, 50 μM 2-mercaptoethanol (2-ME) and 20 ng/ml of human IL-3 (PeproTech). The cell line was authenticated by expression of cell surface markers as previously described [19]. Human primary bone marrow cells were obtained according to Institutional IRB guidelines. Bone marrow mononuclear cells were isolated by Ficoll-paque^™^ plus (GE Healthcare) and maintained in RPMI medium supplemented with 50 μM 2-mercaptoethanol, 1 μM sodium pyruvate and 10% fetal bovine serum (FBS). Human primary cells were obtained in accordance with the Declaration of Helsinki and approval from the institutional review board, protocol # 88-00241. MDS/AML samples were obtained from Leukemia Tissue Bank of the Ohio State University Comprehensive Cancer Center (OSU CCC). MDS/AML cells were thawed and maintained in RPMI medium supplemented with 10% fetal bovine serum and 10 ng/ml hIL-3, GM-CSF and stem cell factor (R&D Systems). All media were purchased from GIBCO-Life Technologies.

### MTT assay

MDS-L (1.5 × 10^5^) or normal human primary bone marrow (1 × 10^6^) cells were treated with increasing concentrations of WFA (0 – 20 μM) in 96 well flat-bottom microtiter plates for 48 h in 0.2 ml of media. Cells were cultured in quadruplicates. Treated cells were incubated with 0.5 mg/ml MTT (3-(4, 5-Dimethylthiazol-2-yl)-2, 5-diphenyltetrazolium bromide) dye (Sigma Aldrich) for 4 h followed by solubilization of formazan salt with acidic isopropanol and spectrophotometric measurements at 560 nm and 690 nm. Optical density variation was corrected by subtracting OD 690 from OD 560 nm values. Media background was subtracted from all treatment groups and a DMSO control group was included in each experiment. IC50 was computed by Graphpad analysis.

### MDS-L xenotransplantation

Animal studies were conducted under an approved protocol (# 2011-0904) by the University of Kentucky Institutional Animal Care and Use Committee. NOD/SCID-IL2R γ-hSCF/hGM-CSF/hIL3 (NSGS) mice were obtained from The Jackson Laboratories (Bar Harbor, ME). 6 -7 month old male and female littermates were exposed to 2.5Gy irradiation in a Mark I-68 ^137^Cesium γ-irradiator (J.L Shepherd and Associates). Four hours later, mice were engrafted by intravenous injection with MDS-L cells (1 × 10^6^ cells/mouse).

Mice were treated from day 14 post-engraftment with 8 mg/kg of WFA intraperitoneally 5x a week for 6 weeks. Control mice received vehicle (10% DMSO, 20% Cremophor-Ethanol (1:3), 70% phosphate PBS). MDS-L engraftment was assessed by the percentage of human CD45^+^/CD33^+^ positive cells in the bone marrow compartment. Positively stained cells were detected by the BD LSRII flow cytometer and the data was analyzed by the FlowJo (Ashland, OR) single cell analysis software.

### Bone marrow histology

Bones which had been preserved in formalin (Fisher Scientific #SF93-4) were washed with running tap water for an hour and decalcified in Richard-Allan scientific decalcifying solution (Thermo Scientific #8340) for 3 h. Decalcified bones were washed, cut horizontally, processed, embedded in paraffin, sectioned and routinely stained with hematoxylin and eosin. The slides were evaluated by a veterinarian blinded to group treatments for abnormalities.

### Mitochondrial membrane potential by JC-1

MDS-L cells (7.5 × 10^5^ cells/ml) were exposed to WFA (10 μM) or DMSO for 8 h. Cells treated with FCCP (50 μM) for 2 h were used as a positive control. JC-1 was added at a 1 μM final concentration to cells for the last 30 min of treatment at 37°C and fluorescence was measured by the iCyt Synergy sorter system (Sony Biotechnology Inc., San Jose, CA) with 488 and 561 nm lasers. WinList 3d 8.0 software (Verity Software House Inc., Topsham, Maine) was used for data analyses. For microscopy, cells treated with WFA (10 μM) or DMSO and stained with JC-1 as described above were mounted on poly-l Lysine (MilliporeSigma-Aldrich # P-6282) coated slides by Cytospin. Pictures were taken on the same day with a Nikon A1RSi confocal microscope (Nikon Instruments Inc, Melville, NY).

### Immunoblotting

MDS-L cells (7.5 × 10^5^ cells/ml) were cultured with 10 μM WFA or DMSO for different time points. Alternatively, cells were pretreated with JNK-IN-8 (10 μM) or NAC (25 mM) for 4 h before further treatment with WFA (10 μM) for the indicated time points. Cells were lysed in Cell Signaling lysis buffer (#9803) containing 1mM PMSF (Sigma P7626), 2mM NaF (Sigma S-1504), 2mM Na_3_VO_4_ (Sigma S-6508) and 1x protease inhibitor cocktail (Roche 5892953001). 35 μg total protein/sample of total lysate was subjected to SDS polyacrylamide gel electrophoresis. Western blot analysis was performed as previously described [51]. Band densitometry analysis was performed using the NIH ImageJ program. Protein expression was normalized to GAPDH, β-actin or total target protein expression as appropriate.

### Annexin-V apoptosis assay

The annexin-V apoptosis detection kit (A432) from Leinco Technologies (St. Louis, MO) was used for annexin-V assays. Thawed human primary MDS/AML cells were maintained in culture for 24 h and then treated with increasing concentrations of WFA for 24 h. Treated cells were stained with annexin-V-FITC and PI following the manufacturer's protocol. Data was acquired with the Becton-Dickinson FACSCalibur flow cytometer and analyzed with the BD CellQuest™ Pro software (San Jose, CA). For MDS-L cells, 7.5 × 10^5^ cells/ml were treated with increasing WFA concentrations for 48 h before staining. In the case of JNK or ROS inhibition studies, 7.5 × 10^5^ MDS-L cells/ml after a 4 h JNK-IN-8 (10 μM) or NAC (25 mM) pretreatment, or no pretreatment were treated with WFA (10 μM) for 24 h and stained with annexin-V/PI. Stained MDS-L cells were detected by the BD LSRII flow cytometer and BD CellQuest™ Pro software was used for data analyses.

### Affymetrix microarray analyses

10 × 10^6^ MDS-L cells were treated with WFA (10 μM) or DMSO for 6 h or 12 h and total RNA was extracted using the Direct-zol^™^ RNA miniprep kit (Zymo Research #R2051). RNA purity was assessed using the Agilent RNA 6000 Nano assay kit (#5067-1511) on the Agilent 2100 Bioanalyzer (Agilent Technologies, Santa Clara, CA). The RNA integrity number (RIN) was ≥ 9 for all samples. Sense-strand DNA (ss-cDNA) was generated, amplified and biotinylated using the WT Plus Reagent kit (Affymetrix, Santa Clara, CA) from 100 ng total RNA per sample. 30 μg of fragmented biotin-labelled ss-cDNA was hybridized to Affymetrix human gene 2.0 ST arrays at 45°C and 60 rpm for 16 h. The arrays were washed, stained using the Affymetrix fluidics station FS 450 and scanned on the Affymetrix 7G GeneChip Scanner. The raw microarray data files were processed through Oligo [52] for data extraction and normalization.

Gene expression profiles of MDS-L cells were examined in triplicate using Affymetrix human gene 2.0 ST arrays. Differential expression analyses comparing WFA-treated and control groups were performed by *limma* [53]. Significantly up/downregulated genes were determined as fold change > 3 and q-value < 0.05. Gene set enrichment analysis was performed using GSEA software and the Hallmark gene sets in the Molecular Signature Database (MSigDB) [54].

### Quantitative real-time PCR (qRT-PCR)

Total RNA was extracted by TRIzol^R^ reagent (LifeTechnologies #15596-018) and cDNA was synthesized from 500 ng of total RNA with qScript reverse transcriptase (Quanta Biosciences #95048-100). iTaq^™^ universal SYBR^R^ green fluorescent Supermix (Biorad #172-5121) was used to quantify mRNA expression. RNA polymerase II was used as an internal control. BIO-RAD CFX Manager software was used to perform relative quantification of target genes using the comparative C_T_ (ΔΔC_T_) method. Primers were obtained from Integrated DNA Technologies (IDT, Coralville, Iowa) and the sequences are provided in Supplementary Table 1.

### ROS measurements

Cell permeant 6-Carboxy-2', 7'-Dichlorodihydrofluorescein Diacetate (Carboxy-H_2_DCFDA) (ThermoFisher Scientific #C400), was used as an indicator for intracellular ROS measurement. MDS-L cells were treated with DMSO, WFA (10 μM) or H_2_O_2_ (1 mM) for 30 min at 37°C. Alternatively, cells were treated with N-acetyl-cysteine (NAC) (25 or 50 mM) for 4 h followed by DMSO, WFA (10 μM) or H_2_O_2_ (1 mM) for additional 30 min at 37°C. Treated cells were suspended in warm H_2_DCFDA solution (1.25 μg/ml in PBS) and incubated in the dark at 37°C for 20 min. Fluorescence was detected on the BD LSR II flow cytometer and the BD CellQuest™ Pro software was used for data analyses. The oxidized form of DCFDA, 5-(and-6)-Carboxy-2', 7'-Dichlorofluorescein Diacetate (Carboxy-DCFDA) (ThermoFisher Scientific #C369), was used as a control for uptake, cellular esterase activity and decay.

### AP-1 luciferase assay

TK-renilla luciferase vector was a generous gift from the laboratory of Dr. Martha Peterson, Department of Microbiology, Immunology and Molecular Genetics, University of Kentucky, USA. 2.5 × 10^6^ cells were co-transfected with 20 μg of TK Renilla luciferase vector and 40 μg of firefly luciferase pGL3 vector (AP-1 or empty vector) at 250 mV, 960 μF and 200 Ω in 200 μl of MDS-L culture medium with a Gene Pulser electroporator (BIO-RAD, Hercules, CA). Transfected cells were cultured in MDS-L culture medium for 24 h at 8.5 × 10^5^ cells per well in 6 well flat bottom plates (Corning #353224). 24 h after transfection, 1 × 10^5^ cells per well were treated with 10 μM WFA or 30 ng/ml PMA for 12 h in white 96 well flat bottom polystyrene plates (Corning #3917, Corning Incorporated Inc, Durham, NC). Promoter activity was assessed by the Dual-Glo^®^ Luciferase assay system (Promega #E2920, Promega Corporation, Madison, WI). Luminescence was measured using the GloMax^®^ Explorer luminometer (Promega). Media background luminescence was subtracted and the ratio of firefly to renilla luminescence was calculated.

### Statistical analysis

Statistically significant differences between groups were evaluated by Student's t test or Tukey's test for *post hoc* pairwise multiple comparisons as appropriate and p values < 0.05 were considered significant. GraphPad Prism 6.05 was used for statistical analyses (GraphPad Software, Inc., La Jolla, CA).

Additional methods are provided in Supplementary Materials.

## SUPPLEMENTARY MATERIALS FIGURES AND TABLES



## Abbreviations

MDS: Myelodysplastic syndromes
AML: Acute myeloid leukemia
WFA: Withaferin A
ROS: Reactive oxygen species
LENA: Lenalidomide
GSEA: Gene set enrichment analysis
FDR: False discovery rate
FWER: Family-wise error rate
MMP: Mitochondrial membrane potential
qRT-PCR: Quantitative Real-Time PCR
NAC: N-acetyl cysteine
DMSO: Dimethyl sulfoxide
FCCP: MTT - 3-(4, 5-Dimethylthiazol-2-yl)-2, 5-diphenyltetrazolium bromide, Trifluoromethoxy)phenylhydrazone
H_2_O_2_: hydrogen peroxide
PMA: Phorbol 12-myristate 13-acetate
DCFDA: 6-Carboxy-2', 7'-Dichlorodihydrofluorescein Diacetate
