# Supplementary material for: Oxidative stress-induced JNK/AP-1 signaling is a major pathway involved in selective apoptosis of myelodysplastic syndrome cells by Withaferin-A
Source: Oncotarget. 2017 Aug 24;8(44):77436–52. doi: 10.18632/oncotarget.20497 (PMC5652791; doi:10.18632/oncotarget.20497)
Supplement: Supplementary file 1 [file oncotarget-08-77436-s001.pdf]

Oxidative stress-induced JNK/AP-1 signaling is a major pathway involved in selective apoptosis of myelodysplastic syndrome cells by Withaferin-A

SUPPLEMENTARY MATERIALS

Cell growth and trypan blue dye exclusion cell viability *assays*

7.5×10^5 MDS-L cells were seeded in 1 ml of media per well in a 12-well plate. The cells were cultured in a humidified atmosphere of 5% CO2 at 37 °C and counted on days 3, 6 and 9. 10 µM lenalidomide or DMSO (0.2%) was added daily during experiments. Cells were split and a seeding density of 7.5×10^5 MDS-L cells/ml was maintained at each time point. Cumulative cell number was calculated from the cell counts at each time point. For trypan blue exclusion, MDS-L cells (7.5×10^5 cells/ml) were treated with increasing concentrations of WFA (0 – 20 µM) in 0.5 ml of media per well of 24 well flat-bottom plates for 48 h and cell viability was assessed by counting live cells by trypan blue exclusion.

Staining and identification of bone marrow stem cells

Bone marrow cells were incubated with normal rat IgG (10 µg/1 × 10^6cells) at 4 °C for 15 min to block Fcγ receptors and then labeled with biotin coupled rat anti-mouse lineage specific antibodies to CD11b (Mac-1), B220, Gr-1, CD8α, Ter-119 and CD5. The cells were stained with c-KIT-APC, Sca-1-PB and streptavidin APC CY7 antibodies for 30 min at 4°C in the dark and washed with 1X FACS buffer. Positively stained cells were detected on the BD LSRII flow cytometer and data was analyzed by the BD CellQuest ™ Pro software. Lineage negative cells which were double positive for both Sca-1 (stem cell antigen-1) and c-KIT (LSK) were identified as hematopoietic stem cells.

NF-κB nuclear translocation

MDS-L cells (5 × 10^6) were incubated with WFA (10 µM) or DMSO for 4 h. The cells were fixed with 70% ethanol for 1 h, blocked for 1 h in 10% normal goat serum and stained with 1:200 dilution of NF-κB p65 primary antibody (Santa Cruz-372), 1:200 DyLight 488 conjugated AffiniPure F(ab')2 goat anti-rabbit secondary antibody (Jackson Immunoresearch) and 1:4000 DAPI (Life Technologies) as described previously by McKenna et al[1]. Slides were viewed and pictures taken on a FV1000 v1.5 confocal microscope (Olympus, Shinjuku, Tokyo, Japan). Cell pellets of MDS-L cells treated with 10 µM WFA or DMSO (0.2%) for 4 h were lysed following the Thermo Scientific Nuclear and Cytoplasmic Extraction kit (#78833) manual. Nuclear and cytoplasmic lysates obtained were analyzed by immunoblotting to determine the sub-cellular distribution of NF-κB p65.

Cell cycle analyses

MDS-L cells (7.5×10^5 cells/ml) were exposed to increasing concentrations of WFA (0 – 5 µM) for 48 h and cell cycle analysis was performed by PI staining as described by McKenna et al [1]. PI fluorescence was detected on the BD FACSCalibur flow cytometer and analyses were performed using the ModFit Software (Verity Software House Inc., Topsham, Maine).

Cell culture

The human AML KG1 cell line was maintained in IMDM medium supplemented with 10% fetal bovine serum.

REFERENCE

1. McKenna MK, Gachuki BW, Alhakeem SS, Oben KN, Rangnekar VM, Gupta RC, Bondada S. Anti-cancer activity of withaferin A in B-cell lymphoma. Cancer Biol Ther. 2015; 16: 1088-98. https://doi.org/10.1080/15384047.2015.1046651.

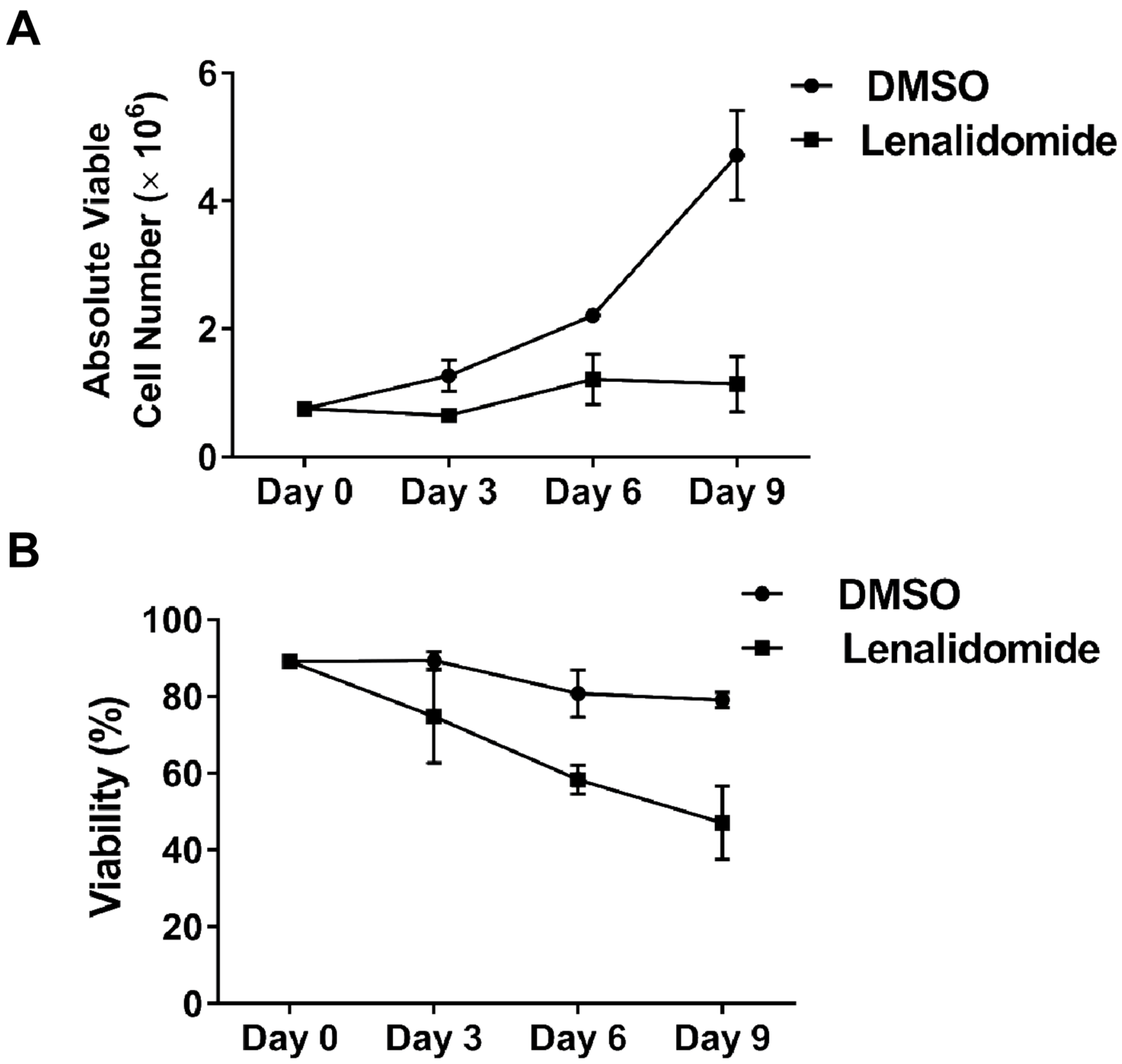

Supplementary Figure 1: Lenalidomide-induced cytotoxicity of MDS-L cells obtained with drug replenishment every 24 h. MDS-L cells were cultured in the presence of DMSO or lenalidomide (10 µM) which were added daily. The number **(A)** and percentage **(B)** of viable cells was determined by trypan blue exclusion on the indicated days. Data are presented as mean ± SD of triplicate cultures. Results are representative of three independent experiments.

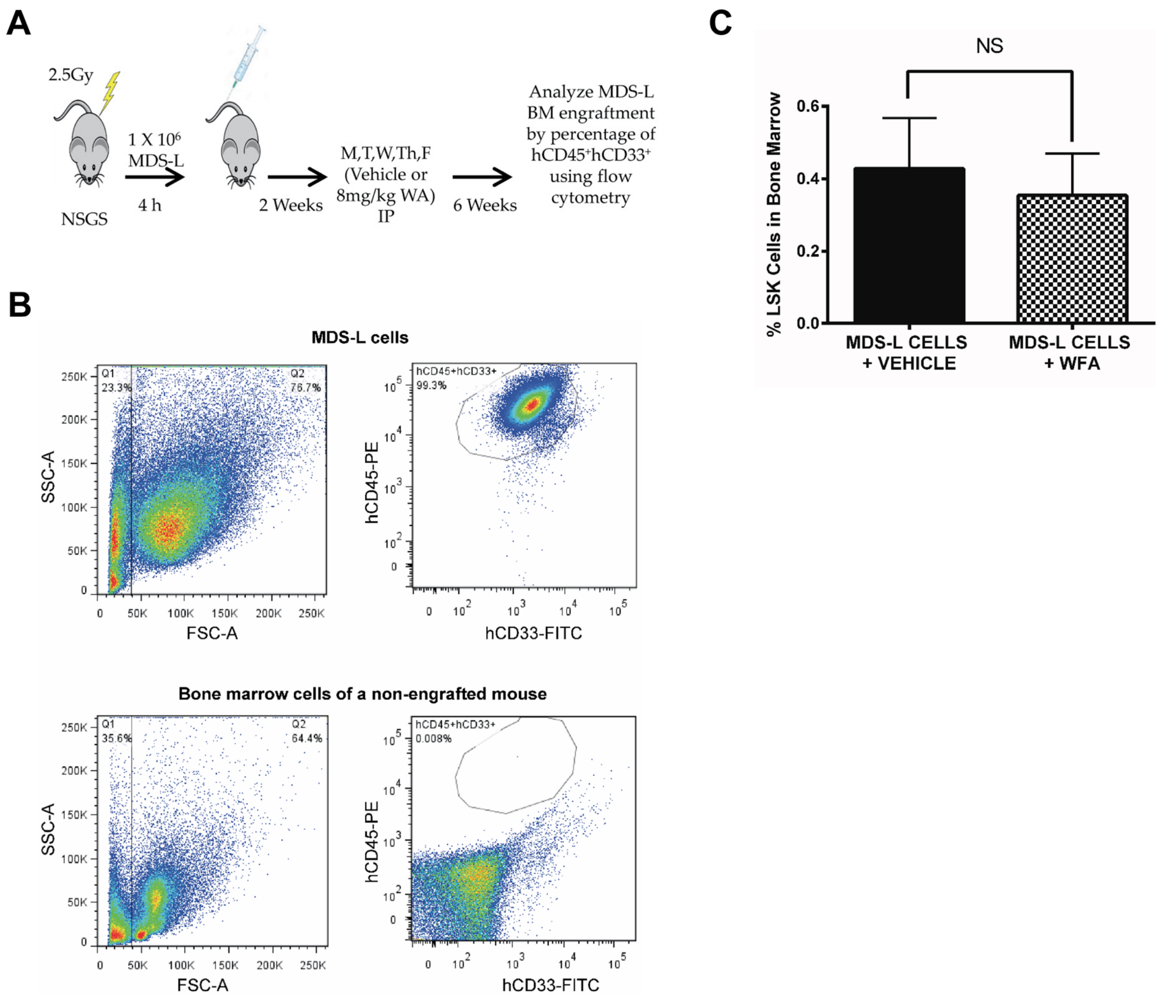

Supplementary Figure 2: MDS-L engraftment in NSGS mice. **(A)** Schematic representation of the experiment conducted to test the *in vivo* effect of WFA in the MDS-L NSGS mice xenograft model. **(B)** Flow cytometry gating scheme used for positive and specific identification of engrafted human MDS-L cells. Accuracy and specificity was ensured by the absence of false positive cells in non-engrafted mice. **(C)** WFA does not suppress endogenous mouse bone marrow stem cells. Bone marrow cells from WFA or vehicle treated engrafted mice were labeled with biotin-coupled rat anti-mouse lineage specific antibodies to CD11b (Mac-1), B220, Gr-1, CD8α, Ter-119 and CD5. Lineage negative, Sca-1 positive and c-KIT positive (LSK) stem cells were identified by flow cytometry analyses of lineage labelled cells stained with streptavidin APC CY7, Sca-1-PB and c-KIT-APC. Mean % of LSK cells for a minimum of 5 mice per group ± SD is shown.

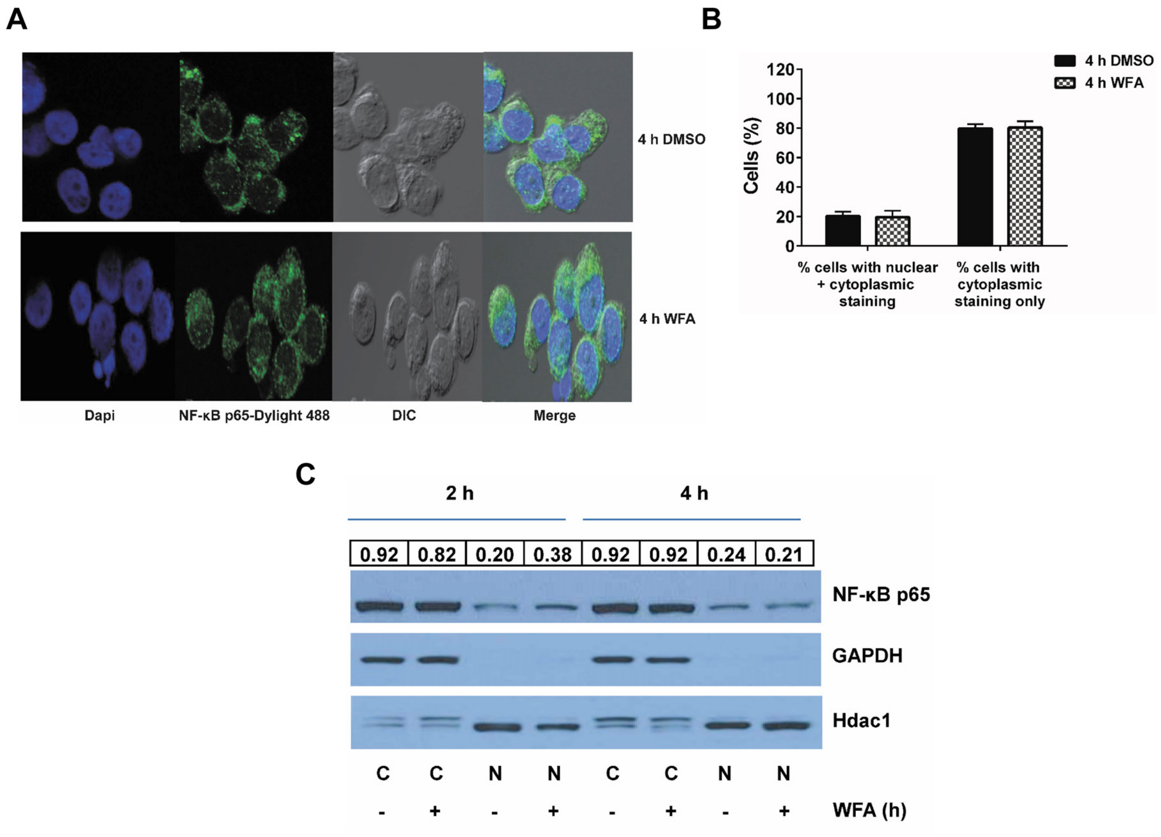

Supplementary Figure 3: Cytotoxic effects of WFA in MDS-L cells are independent of NF-κB activation. (A) MDS-L cells treated with 10 μM WFA or DMSO for 4 h were stained for the p-65 subunit of NF-κB; the nuclear and cytoplasmic distribution of NF-κB was determined by fluorescence microscopy. A representative field is shown with individual interference microscopy (DIC), fluorescence images and the merged images. **(B)** Quantification of cells (> 200 cells) from several fields with nuclear and/ or cytoplasmic p-65 staining in MDS-L cells treated with10 μM WFA or DMSO for 4 h. **(C)** Nuclear and cytoplasmic protein fractions from cells treated with 10 μM WFA or DMSO for 2 or 4 h were analyzed by western blot to determine the effect of WFA treatment on nuclear translocation of the p65 subunit of NF-κB. Cytoplasmic (C) or nuclear (N) p65 NF-κB was normalized to GAPDH or Hdac1 respectively; values in boxes are the densitometric ratios. Data are representative of two independent experiments.

6 h

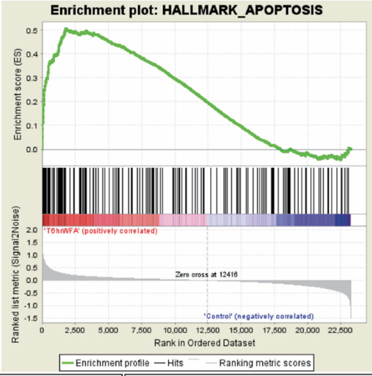

Dataset	gsea.Control_vs_T6hrWFA.cls #T6hrWFA_versus_Control.Control_vs_T6hrWFA.cls #T6hrWFA_versus_Control_repos
Phenotype	Control_vs_T6hrWFA.cls#T6hrWFA_versus_Control_repos
Upregulated in class	T6hrWFA
GeneSet	HALLMARK_APOPTOSIS
Enrichment Score (ES)	0.50754654
Normalized Enrichment Score (NES)	2.0332267
Nominal p-value	0.0
FDR q-value	0.0
FWER p-Value	0.0

12 h

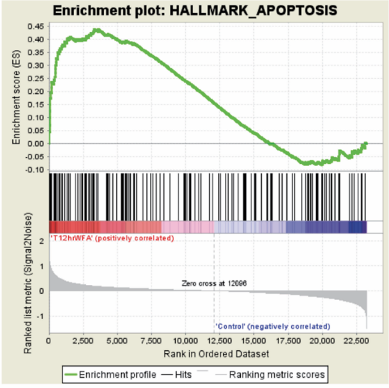

Dataset	gsea.Control_vs_T12hrWFA.cls #T12hrWFA_versus_Control.Control_vs_T12hrWFA.cls #T12hrWFA_versus_Control_repos
Phenotype	Control_vs_T12hrWFA.cls#T12hrWFA_versus_Control_repos
Upregulated in class	T12hrWFA
GeneSet	HALLMARK_APOPTOSIS
Enrichment Score (ES)	0.4382375
Normalized Enrichment Score (NES)	1.7445616
Nominal p-value	0.0
FDR q-value	0.0016486881
FWER p-Value	0.008

0 = 0.0001

Supplementary Figure 4: Genes differentially regulated by WFA are linked to apoptosis induction. Gene set enrichment analysis (GSEA) revealed enrichment of apoptosis-related genes with WFA treatment, at both 6h and 12h, compared to control. In the table the nomral and FWER p-values as well as the FDR q-values were 0.0001 but were rounded to zero by the GSEA software.

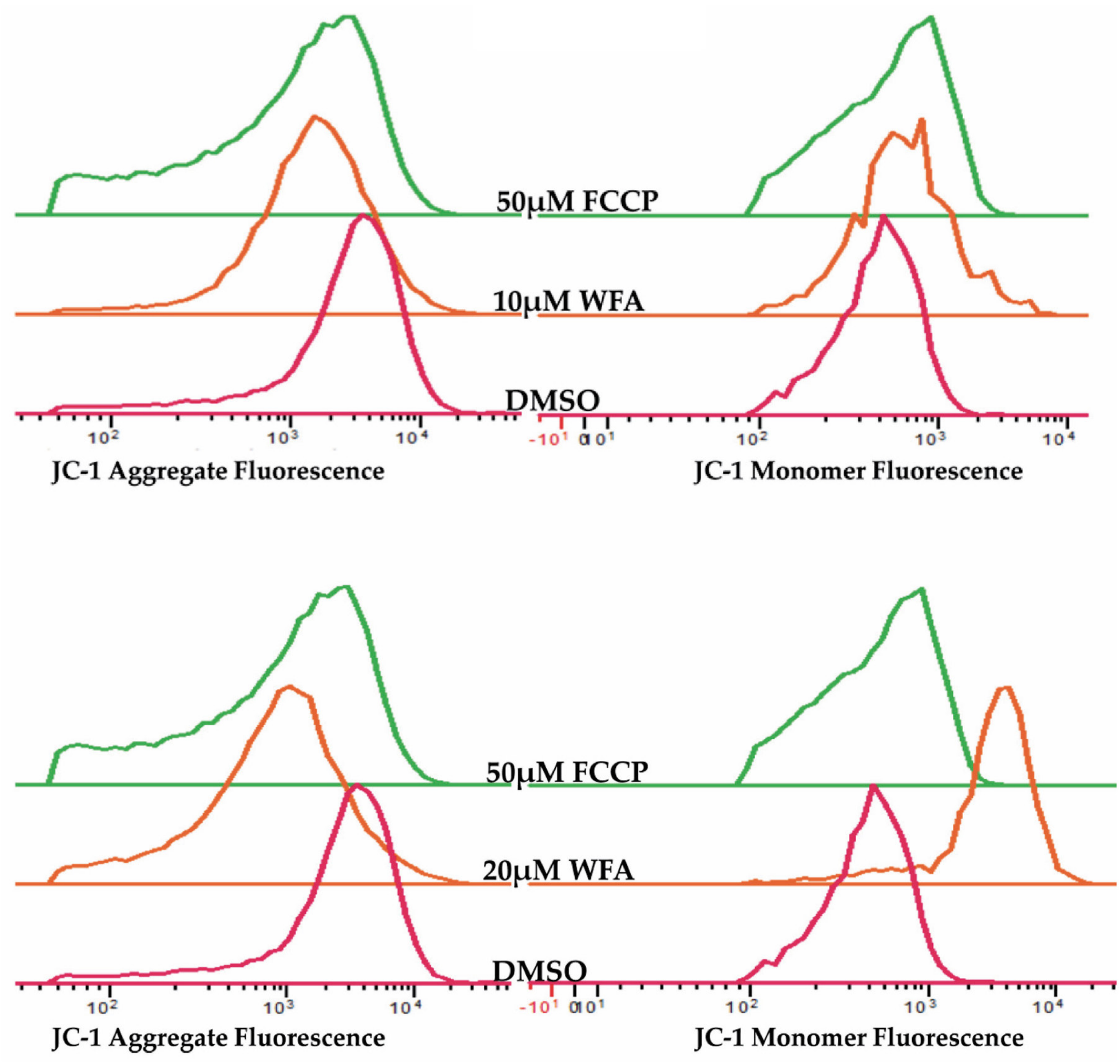

Supplementary Figure 5: WFA decreased mitochondrial membrane potential in MDS-L cells. Representative overlays of JC-1 aggregates or monomers by mean fluorescent intensity. MDS-L cells were treated with WFA (10 μM or 20 μM) for 8 h or an inhibitor of mitochondrial oxidative phosphorylation, FCCP, (50 μM) for 2 h. JC-1 (1 μM) was added during the last 30 min of treatment and the distribution of JC-1 monomers and aggregates were analyzed by flow cytometry.

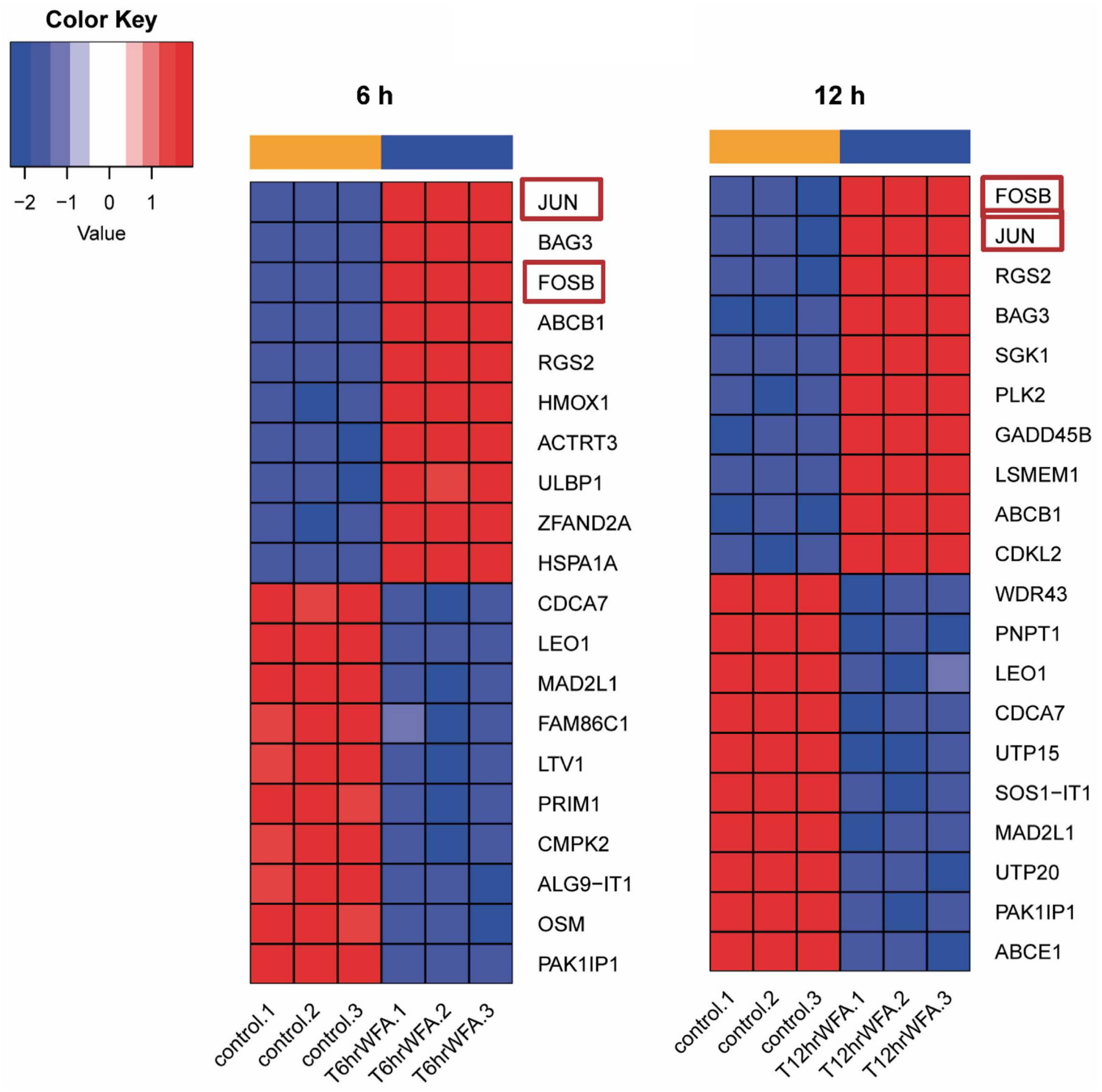

Supplementary Figure 6: Heatmap of top 20 WFA regulated genes by fold change. The top 10 increased (top half) and repressed (bottom half) genes obtained with WFA treatment at 6 h and 12 h arranged by fold change are shown.

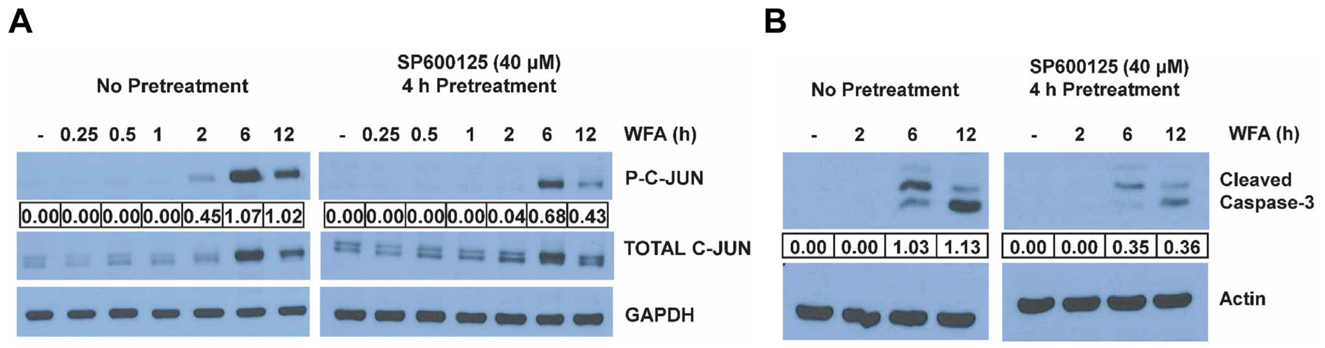

Supplementary Figure 7: SP600125 inhibits WFA-induced JNK and caspase-3 activation. MDS-L cells pretreated with SP600125 (40 μM) for 4 h or without pretreatment were exposed to WFA for an additional 0.25, 0.5, 1, 2, 6, or 12 h. JNK activation by P-c-Jun expression **(A)** and caspase-3 activation by cleaved caspase-3 expression **(B)** were assessed by immunoblot.

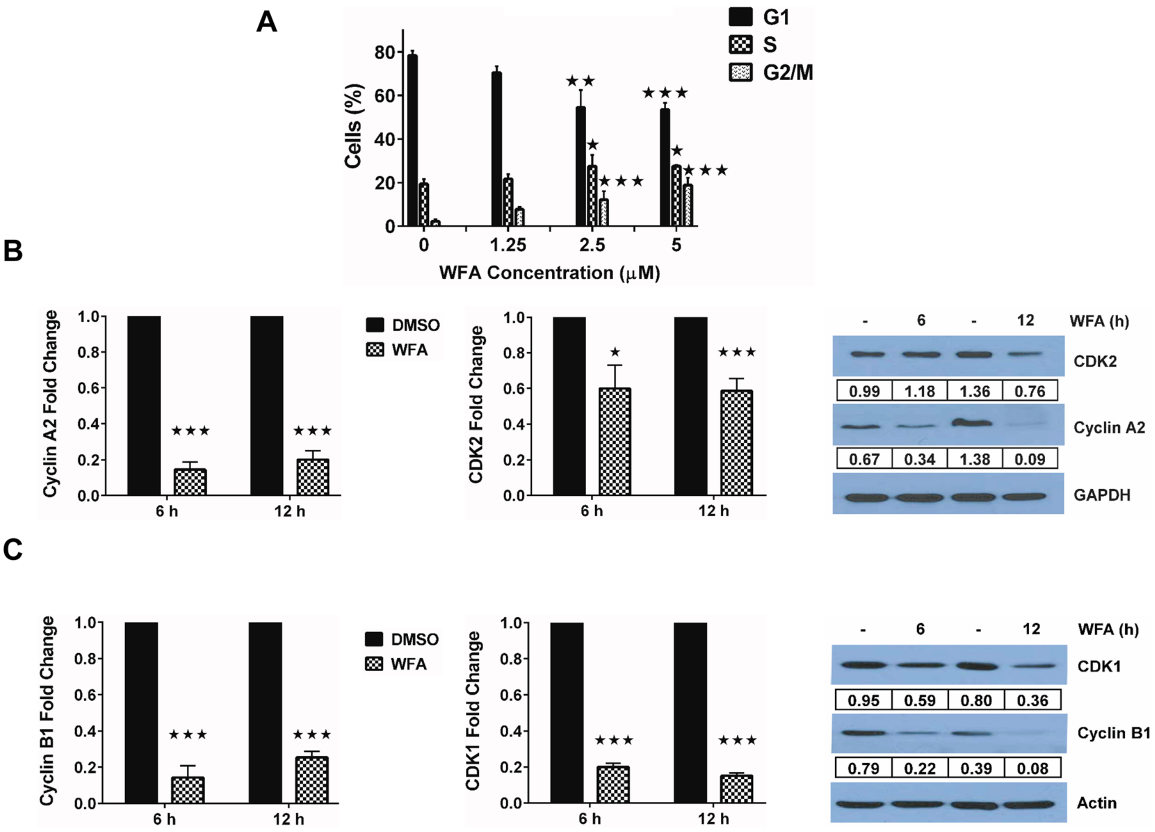

Supplementary Figure 8: WFA treatment induces cell cycle arrest in MDS-L cells. (A) Cells were treated with the indicated concentrations of WFA for 48 h and stained with propidium iodide (PI). PI staining intensity, which correlates with DNA content, was analyzed by flow cytometry. The fraction of cells (%) at each phase is shown. Data are presented as mean ± SD of triplicate cultures. Total RNA and whole cell lysates from control or WFA treated (6 or 12 h) MDS-L cells were analyzed by qRT-PCR and immunoblot, respectively, for cyclin A and CDK2 **(B)** and cyclin B and CDK1 **(C)** expression. Gene amplification was normalized to RPII expression and relative amplification was determined by normalizing to DMSO control values . * = $p<0.05$, *** = $p<0.0005$. Results are representative of two independent experiments.

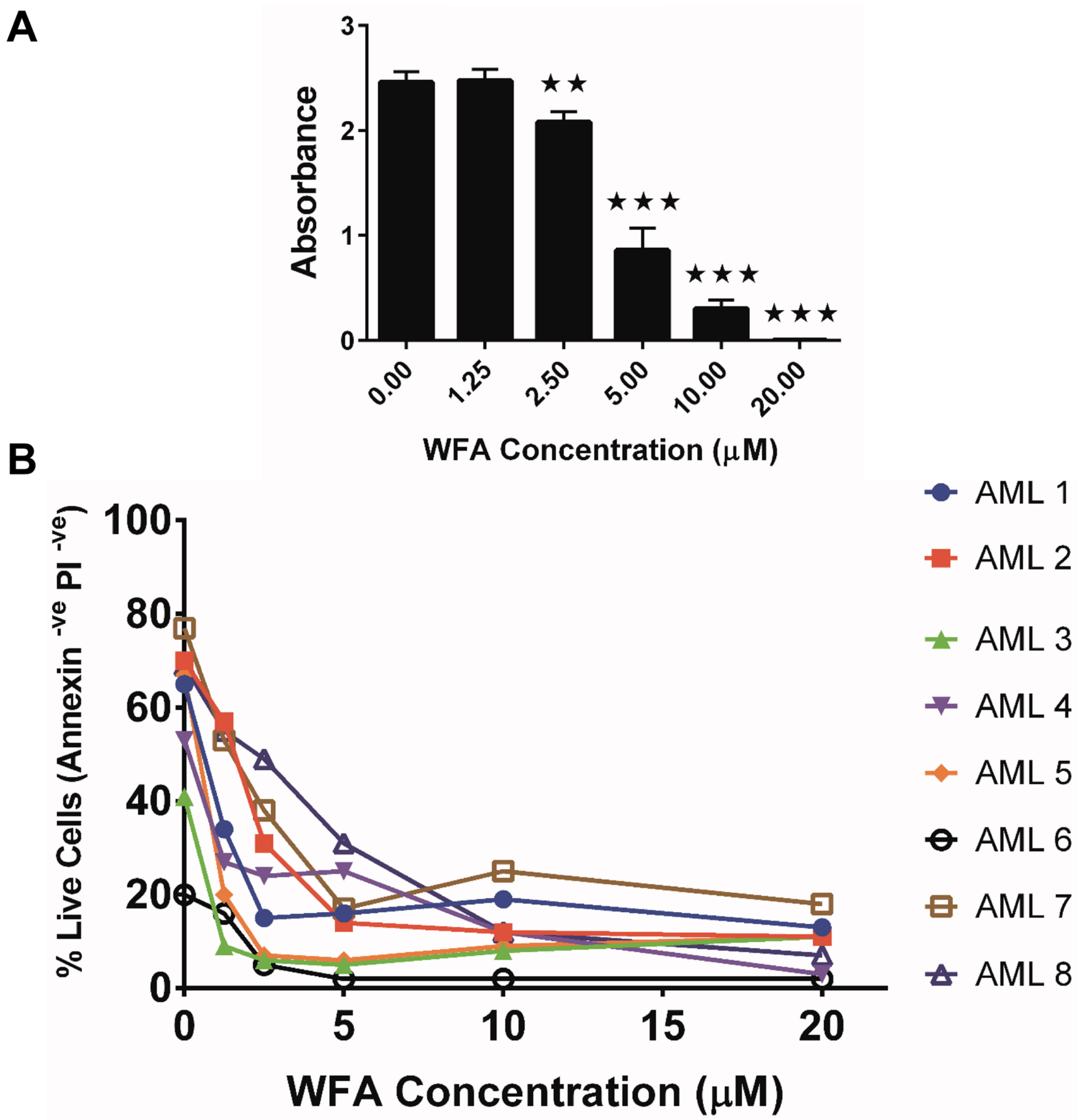

Supplementary Figure 9: WFA is cytotoxic to AML cells. **(A)** WFA significantly decreased the viability of KG1 AML cells *in vitro*. KG-1 cells were treated with increasing concentrations of WFA for 48 h and cell viability was assessed by MTT. Data are presented as mean ± SD of triplicate cultures. * = p<0.05 and *** = p<0.0005 indicate statistically significant differences between absorbance values obtained with WFA and DMSO control treatments. Results are representative of two independent experiments. **(B)** Cell viability of human primary AML samples assessed by annexin-V/PI staining for apoptosis. AML patient-bone marrow cells were treated with various concentrations of WFA for 24h and stained with annexin-V and PI. The percentage of live or non-apoptotic cells defined as annexin-V/PI negative are shown.

Supplementary Table 1: List of qRT-PCR primers

Gene		Primers
BAG3	Forward	5'-GGAGATCAAGATCGACCCGC-3'
	Reverse	5'-CAGAGGATGGAGTCTCCTTGG-3'
RGS2	Forward	5'-TTCAACACGACTGCAGACCC-3'
	Reverse	5'-CTTCCTCAGGAGAAGGCTTGAT-3'
JUN	Forward	5'-GCCAACTCATGCTAACGCAG-3'
	Reverse	5'-GGCAGGCCAGAAAGAGTTCA-3'
FOSB	Forward	5'-GCGTACTTTGAGGACTCGCT-3'
	Reverse	5'-TTCCTCTGGGGTGAGCGTCT-3'
BCL2L11 (BIM)	Forward	5'-ACCAGATCCCCGCTTTTCAT-3'
	Reverse	5'-GAAGAGGCATCCTCCTTGCATA-3'
CDKN1A (P21)	Forward	5'-AGTACCCTCTCAGCTCCAGG-3'
	Reverse	5'-TGTCTGACTCCTTGTTCCGC-3'
CCNA2 (CYCLIN A2)	Forward	5'-TCGCGGGATACTTGAACTGC-3'
	Reverse	5'-GTGCAACCCGTCTCGTCTT-3'
CDK2	Forward	5'-GGATGCCTCTGCTCTCACTG-3'
	Reverse	5'-ACAGGGTCACCACCTCATGG-3'
CCNB1 (CYCLIN B1)	Forward	5'-GCCTGAGCCTATTTTGGTTGA-3'
	Reverse	5'-AGTGACTTCCCGACCCAGTA-3'
CDK1	Forward	5'-AAGCCGGGATCTACCATACC-3'
	Reverse	5'-GCTCTGGCAAGGCCAAAATC-3'
RPOL2	Forward	5'-GCACCACGTCCAATGACAT-3'
	Reverse	5'-GTGCGGCTGCTTCCATAA-3'
